# Relationship Between *Helicobacter pylori*, Trimethylamine‐N‐Oxide, and Carotid Plaque Formation Among Adult Health Checkup Population in China

**DOI:** 10.1155/crp/5468109

**Published:** 2026-10-08

**Authors:** Fang Liu, Lina Zheng, Xia Cao, Yaoze Li, Xiangdong Xu, Yihan Zhou, Yue Wang, Yun Zhang, Xue Sun, Pei Peng, Jiangman Zhao

**Affiliations:** ^1^ Department of Internal Medicine Kaifeng Central Hospital Kaifeng 475000 Henan China; ^2^ Department of Neurosurgery Queshan County People’s Hospital Queshan 463200 Henan China; ^3^ Department of Neurology Queshan County People’s Hospital Queshan 463200 Henan China; ^4^ Shanghai Biotecan Pharmaceuticals Co., Ltd. Shanghai 201204 China; ^5^ Shanghai Zhangjiang Institute of Medical Innovation Shanghai 201204 China; ^6^ Shanghai Biotecan Medical Laboratory Co., Ltd Shanghai 201204 China; ^7^ Department of General Medicine Kaifeng Central Hospital Kaifeng 475000 Henan China

**Keywords:** carotid plaque formation, *Helicobacter pylori*, trimethylamine-N-oxide

## Abstract

**Background:**

Both trimethylamine‐N‐oxide (TMAO) and *Helicobacter pylori* (*H. pylori*) have been linked to atherosclerosis and carotid plaque development. This study examined the associations of *H. pylori* infection and plasma TMAO levels with carotid plaque in a health screening population.

**Methods:**

Between October 2022 and September 2023, 1779 participants undergoing routine health examinations, including carotid ultrasonography and *H. pylori* testing, were enrolled. Multivariable logistic regression was used to evaluate the association between *H. pylori* infection and carotid plaque. Inverse probability weighting (IPW) was applied to address selection bias in the TMAO subgroup, followed by weighted logistic regression analyses.

**Results:**

Carotid plaque was more prevalent among *H. pylori*‐seropositive participants than among seronegative participants (53.96% vs. 40.37%, *p* < 0.001). In multivariable logistic regression analyses, *H. pylori* seropositivity remained significantly associated with carotid plaque after adjustment for demographic characteristics and cardiovascular risk factors, with odds ratios ranging from 3.099 to 3.392 across the adjusted models (all *p* < 0.001). Higher TMAO levels were associated with carotid plaque in unadjusted analyses and remained associated after adjustment for demographic and conventional cardiovascular risk factors; however, these associations were attenuated and became statistically non‐significant after further adjustment for age. No significant association was observed between *H. pylori* seropositivity and plasma TMAO levels.

**Conclusions:**

*H. pylori* infection was associated with carotid plaque in this cross‐sectional study. The association between TMAO and carotid plaque appeared to be largely explained by age. Given the cross‐sectional design, causal relationships cannot be inferred, and further prospective studies are warranted.

## 1. Introduction

Cardiovascular diseases (CVDs), a group of disorders affecting the heart and blood vessels, represent one of the leading causes of global mortality and morbidity [1]. In China, CVDs are responsible for approximately 40% of all deaths, with mortality rates exceeding those of cancer and other major diseases [2]. Since 2006, the prevalence of CVDs has shown a persistent upward trend, characterized by an earlier disease onset, a shift largely attributed to the rapid socioeconomic transformation in modern society. Atherosclerosis, characterized by arterial wall thickening, subendothelial lipid deposition, and plaque formation, constitutes the fundamental pathological process underlying most CVDs [3, 4]. This process is driven not only by well‐established risk factors such as advanced age, hypertension, smoking, obesity, dyslipidemia, diabetes, and genetic predisposition but also by emerging risk factors including *Helicobacter pylori* (*H. pylori*) infection and elevated levels of trimethylamine‐N‐oxide (TMAO) [5–7].

*H. pylori*, a Gram‐negative bacterium, is a well‐established etiological agent in various gastrointestinal disorders, including chronic gastritis, peptic ulcer disease, and gastric carcinoma. While *H. pylori* does not directly induce pathology, persistent infection causes chronic inflammation of the gastric mucosa, which is central to the pathogenesis of these conditions [8]. Beyond its canonical role in gastrointestinal diseases, growing evidence indicates that *H. pylori* infection may exert systemic effects. Recent studies suggest a potential link between chronic *H. pylori* infection and an increased risk of CVDs, with inflammation and immune dysregulation triggered by the infection posited as key mediators in CVD pathogenesis [9]. Notably, *H. pylori* infection has been associated with a heightened risk of atherosclerosis [10]. A meta‐analysis further indicated that infected individuals under 60 years of age and without conventional cardiovascular risk factors face a greater likelihood of developing atherosclerotic lesions [11].

TMAO, a gut microbiota‐derived metabolite, is increasingly recognized as a significant contributor to the development of CVDs [12]. Elevated plasma TMAO levels have been consistently associated with the progression of atherosclerosis [13]. Murine studies further indicate that TMAO can enhance the viability and virulence of *H. pylori*, thereby exacerbating gastric inflammation and advancing related pathology [14]. Consequently, TMAO may potentiate both the inflammatory response and microbial dysbiosis triggered by *H. pylori* infection. Elucidating the common risk factors linking *H. pylori* infection to carotid plaque formation is essential for understanding the indirect mechanisms through which this infection may promote atherosclerosis. Therefore, this study aimed to investigate the associations between *H. pylori* infection, circulating TMAO levels, and the risk of carotid plaque formation.

## 2. Materials and Methods

### 2.1. Study Populations

A total of 1779 individuals who underwent health examinations that included *H. pylori* testing at Kaifeng Central Hospital were initially screened, as the baseline cohort. The study protocol was approved by the Ethics Committee of Kaifeng Central Hospital (2022ky‐024), and written informed consent was obtained from all participants. Enrollment was based on predefined inclusion and exclusion criteria. Inclusion criteria comprised availability of complete anthropometric measurements, confirmed *H. pylori* infection status, and comprehensive blood biochemical profiles. Exclusion criteria were as follows: (1) age < 18 years; (2) history of major surgery; and (3) incomplete clinical data. From this baseline cohort, participants were invited to voluntarily undergo TMAO testing. Those who provided an additional fasting blood sample formed the TMAO subcohort (*n* = 728). Baseline characteristics were compared between participants with and without TMAO measurements, as detailed in Supporting Table 1. The participant selection process is illustrated in Figure 1.

**FIGURE 1:**
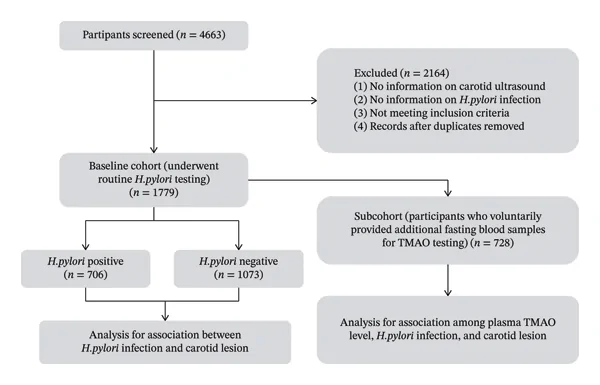
Flowchart of participants’ inclusion.

### 2.2. Data Collection

Baseline demographic and clinical data were collected, including smoking status, alcohol consumption, body mass index (BMI), waist‐to‐hip ratio (WHR), dietary habits (preference for red meat), medical history, and laboratory parameters. Smoking was defined as smoking ≥ 3 cigarettes per day for at least 1 year. Alcohol consumption was defined as drinking at least once per month. Hypertension was diagnosed as systolic blood pressure (SBP) ≥ 140 mmHg, diastolic blood pressure (DBP) ≥ 90 mmHg, and/or current use of antihypertensive medication. BMI was calculated as weight in kilograms divided by height in meters squared (kg/m^2^). Abdominal obesity was defined as WHR > 0.90 for men and > 0.85 for women. Red meat consumption was documented based on participant self‐report. Laboratory assessments included complete blood count, lipid profile, liver function, thyroid function, and renal function parameters. Carotid plaque was identified by ultrasonography and defined per the current Mannheim Consensus criteria as the presence of a focal structure meeting any of the following ultrasound criteria: a carotid intima‐media thickness (cIMT) measurement ≥ 1.5 mm, a focal protrusion increasing local cIMT by ≥ 0.5 mm, or an area where cIMT exceeded that of the adjacent vessel wall by 50% or more [15]. Carotid ultrasonography was performed by trained sonographers using a standardized protocol, Mannheim consensus criteria, and the same ultrasound equipment.

### 2.3. *H. pylori* Infection Detection

*H. pylori* infection status was assessed using a commercial protein chip assay (*Helicobacter pylori* IgG Antibody Detection Kit; Xinkang Gene Digital Technology, Taizhou, China). According to the manufacturer’s protocol, 20 μL of serum from each participant was applied to a nitrocellulose membrane precoated with 3 *H. pylori*‐specific antigens (CagA, VacA, and Ure). Bound IgG antibodies were detected qualitatively using the designated XK2100 biochip reader (Xinkang Gene Digital Technology). A sample was classified as seropositive if the signal intensity for any one of the three target antigens reached or exceeded the predefined cutoff value. As per the manufacturer’s specification, a positive result indicates either current active infection or prior exposure to *H. pylori*. It should be noted that serological testing cannot distinguish between current and past infection, which may introduce nondifferential misclassification.

### 2.4. TMAO Detection

Plasma TMAO levels were quantified exclusively in participants who voluntarily provided an additional fasting blood sample during their routine health examination. These samples were processed immediately after collection to isolate plasma, which was then stored at −80°C until analysis. TMAO concentration was determined using liquid chromatography–tandem mass spectrometry (LC–MS/MS) with an internal standard (TMAO‐d9). Briefly, 200 μL of plasma was protein‐precipitated with 650 μL of acetonitrile containing 80 ng/mL TMAO‐d9, followed by centrifugation at 1000 × *g* for 3 min. The resulting supernatant was collected, dried under a gentle stream of nitrogen, and the residue was reconstituted in 200 μL of deionized water; 160 μL of this solution was injected for analysis. Chromatographic separation was performed on an ACQUITY UPLC I‐Class system (Waters, USA) coupled with a Xevo TQD mass spectrometer, using an ACQUITY UPLC HSS T3 column (2.1 × 100 mm). The mobile phase consisted of 0.1% aqueous formic acid (A) and methanol (B), delivered at a flow rate of 0.3 mL/min with the following gradient: 2% B at 0–1.0 min, increased to 50% B by 2.5 min, and returned to 2% B at 5.0 min. The injection volume was 10 μL. Mass detection was conducted in positive electrospray ionization mode under optimized parameters: capillary voltage, 3.5 kV; cone voltage, 30 V; collision energy, 18 V; desolvation temperature, 350°C; and desolvation gas flow, 650 L/h. Quantification was based on selected reaction monitoring of the mass transitions *m/z* 76⟶58 for TMAO and *m/z* 85⟶66 for the internal standard TMAO‐d9.

### 2.5. Covariate Selection

To minimize potential confounding effects, covariates were selected a priori based on established clinical and epidemiological evidence. The adjustment model included demographic characteristics (age, sex), anthropometric measures (BMI), behavioral factors (smoking, alcohol consumption), clinical comorbidities (hypertension, diabetes), and comprehensive laboratory parameters—including complete blood count, lipid profiles, and other atherogenesis‐related biochemical indices. Although key determinants of TMAO and atherosclerosis, such as detailed dietary intake, antibiotic exposure, and lipid‐lowering medication use, were not comprehensively available, we partially accounted for these factors using surrogate variables including red meat consumption and renal function markers (serum creatinine and BUN).

### 2.6. Statistical Analysis

A complete‐case approach was adopted for all analyses. All statistical analyses were performed using IBM SPSS Statistics, Version 23 (IBM, NY, USA). Continuous variables were presented as mean ± standard deviation or median (interquartile range), depending on their distribution, whereas categorical variables were expressed as frequencies (percentages). Comparisons between groups were performed using the independent‐samples *t*‐test, Mann–Whitney *U* test, analysis of variance (ANOVA), Kruskal–Wallis test, or *χ* ^2^ test, as deemed appropriate.

To investigate the association between *H. pylori* infection and carotid plaque, we first performed univariate logistic regression to screen candidate variables (entry criteria: *p* < 0.10). Subsequently, multivariable logistic regression models were constructed using a stepwise adjustment approach. Model 1 included *H. pylori* infection alone. Model 2 additionally adjusted for demographic factors (age, sex, smoking status, alcohol consumption). Model 3 further adjusted for traditional cardiovascular risk factors (BMI, hypertension, FBG, LDL‐C, HDL‐C, and TG). Model 4 incorporated additional candidate variables that had a *p* < 0.10 in the univariate analysis. For each model, odds ratios (ORs) with 95% confidence intervals (CIs) were reported. Model discrimination was assessed using the area under the receiver operating characteristic curve (AUC), while model calibration was evaluated via the Hosmer–Lemeshow test. Multicollinearity was assessed by calculating variance inflation factors (VIFs), where a VIF < 5 indicated no significant collinearity. To evaluate model parsimony and avoid overfitting, we calculated the events‐per‐variable (EPV) ratio, with a threshold of ≥ 10:1 considered acceptable.

Given the significant differences in key variables between the TMAO subgroup and the subgroup without TMAO measurements, we applied inverse probability weighting (IPW) to correct for selection bias. A propensity score model was constructed and IPW weights were computed as 1/propensity score for the treatment group (with TMAO measurements) and 1/(1‐propensity score) for the control group. Extreme weights were truncated at the 99th percentile. Covariate balance before and after weighting was assessed using standardized mean differences (SMDs), where an SMD < 0.1 indicated satisfactory balance.

In the weighted TMAO subgroup, we performed weighted logistic regression to assess the association between TMAO levels and carotid plaque. TMAO levels were categorized into quartiles, and multivariable weighted logistic regression models were constructed. ORs, 95% CIs, AUC values, and Hosmer–Lemeshow test *p* values were reported for all weighted regression models. All hypothesis tests were two‐tailed, with a *p* < 0.05 considered statistically significant.

## 3. Results

### 3.1. Baseline Characteristics of Participants

Overall, 1779 participants were enrolled, of whom 728 (40.9%) underwent TMAO measurement (TMAO subgroup). The participant selection process is illustrated in Figure 1, and baseline characteristics stratified by *H. pylori* infection status are presented in Table 1. Compared with *H. pylori*‐negative participants, those who were seropositive had a higher proportion of alcohol drinkers, elevated white blood cell, lymphocyte, and neutrophil counts, increased serum creatinine levels, and a notably higher prevalence of carotid artery plaques (all *p* < 0.05).

**TABLE 1:** Baseline characteristics of the study population stratified by *H. pylori* infection status.

	*H. pylori* negative (*n* = 706)	*H. pylori* positive (*n* = 1073)	*p*
Age, years	42.00 (25.00)	44.00 (22.00)	0.202
Male (%)	261.00 (36.97%)	440.00 (41.01%)	0.092
BMI (kg/m^2^)	23.59 (4.78)	23.58 (4.47)	0.603
WHR	0.84 (0.12)	0.84 (0.12)	0.607
Smoker (%)	68.00 (9.63%)	96.00 (8.95%)	0.625
Alcohol drinker (%)	109.00 (15.44%)	218.00 (20.32%)	0.009
Hypertension	148.00 (21.00%)	192.00 (17.90%)	0.107
Diabetes	13.00 (1.80%)	24.00 (2.20%)	0.568
WBC (× 10^9^/L)	5.63 (1.98)	5.81 (2.03)	0.001
Lymphocyte (× 10^9^/L)	1.88 (0.71)	1.90 (0.69)	0.039
Neutrophil (× 10^9^/L)	3.30 (1.53)	3.44 (1.48)	< 0.001
BAS%	0.30 (0.30)	0.30 (0.30)	0.681
EOS%	1.60 (1.70)	1.60 (1.63)	0.982
NLR	1.77 (0.87)	1.80 (0.91)	0.238
LMR	7.20 (3.26)	7.32 (3.32)	0.230
ESR (mm/h)	4.00 (6.00)	4.00 (6.00)	0.371
TG (mmol/L)	1.11 (0.89)	1.12 (0.88)	0.550
TC (mmol/L)	4.57 (1.19)	4.58 (1.20)	0.457
HDL‐C (mmol/L)	1.41 (0.42)	1.40 (0.41)	0.054
LDL‐C (mmol/L)	2.72 (1.00)	2.76 (0.97)	0.083
GGT (U/L)	17.50 (16.00)	17.00 (14.00)	0.458
ALT (U/L)	15.00 (12.00)	16.00 (12.00)	0.171
TBIL (umol/L)	10.60 (5.90)	10.70 (6.30)	0.746
Scr (μmol/L)	62.00 (17.00)	64.00 (18.00)	0.041
BUN (mmol/L)	4.20 (1.40)	4.30 (1.50)	0.950
UA (umol/L)	278.00 (108.50)	282.00 (108.00)	0.886
Plaque formation	285.00 (40.37%)	579.00 (53.96%)	< 0.001

*Note:* WHR, ratio of waist‐to‐hip circumference; BAS%, basophil percentage; EOS%, eosinophil percentage; NLR, neutrophil‐to‐lymphocyte ratio; LMR, lymphocyte‐to‐monocyte ratio; TG, triglycerides; GGT, *γ*‐glutamyl transpeptidase; ALT, alanine transaminase; TBIL, total bilirubin; Scr, serum creatinine.

Abbreviations: BUN, blood urea nitrogen; ESR, erythrocyte sedimentation rate; HDL‐C, high‐density lipoprotein cholesterol; *H. pylori, Helicobacter pylori*; LDL‐C, low‐density lipoprotein cholesterol; TC, total cholesterol; UA, uric acid; WBC, white blood cell.

To address potential selection bias, we compared participants with and without TMAO measurement (Supporting Table 1). Participants in the TMAO subgroup exhibited older age, lower BMI, and a reduced prevalence of hypertension while demonstrating a higher prevalence of diabetes, elevated levels of triglycerides and LDL‐C, as well as an increased incidence of plaque formation (all *p* < 0.05). These differences indicated significant selection bias, which necessitated IPW adjustment.

### 3.2. Association Between *H. pylori* Infection and Carotid Plaque

In the full sample (*n* = 1779), *H. pylori* infection was significantly associated with carotid plaque in univariate analysis (OR 1.731, 95% CI 1.428–2.099, *p* < 0.001; Supporting Table 2). This association remained statistically significant across multivariable models after adjustment for demographic characteristics and traditional cardiovascular risk factors (Table 2). These findings indicate a robust association between *H. pylori* infection and carotid plaque within this study population. The model exhibited good discriminative ability (AUC 0.937, 95% CI 0.924–0.950) and statistically acceptable calibration (Hosmer–Lemeshow *p* = 0.008). No significant multicollinearity was observed (all VIF < 5; Supporting Table 3).

**TABLE 2:** Association between *H. pylori* infection and carotid plaque formation.

Model	OR (95% CI)	*p* ^∗^	AUC (95% CI)	H–L *p* ^#^	EPV
Crude model	1.731 (1.428, 2.099)	< 0.001	0.565 (0.539, 0.592)	< 0.001	—
Multivariable Model 1	3.255 (2.396, 4.422)	< 0.001	0.921 (0.909, 0.934)	0.055	172.8
Multivariable Model 2	3.099 (2.477, 3.876)	< 0.001	0.925 (0.912, 0.937)	0.043	78.5
Multivariable Model 3	3.392 (2.555, 4.502)	< 0.001	0.937 (0.924, 0.950)	0.008	54.0

*Note:* AUC, area under the receiver operating characteristic curve; H–L, Hosmer–Lemeshow. Crude model: Odds ratios and 95% CI were estimated by *H. pylori* infection. Multivariable Model 1 was adjusted for demographic factors (age, sex, smoking status, alcohol consumption). Multivariable Model 2 was additionally adjusted for traditional cardiovascular risk factors (BMI, hypertension, FBG, LDL‐C, HDL‐C, and TG). Multivariable Model 3 was additionally adjusted for additional candidate variables that had a *p* < 0.10 in the univariate analysis (NLR, ESR, GGT, TBIL, SCR). *p* ^∗^ < 0.05 were considered statistically significant. H‐L *p* ^#^ > 0.05 indicates good model calibration.

Abbreviations: CI, confidence interval; OR, odds ratio.

### 3.3. IPW and Covariate Balance

To address selection bias, we estimated propensity scores for TMAO measurement status and computed IPW weights. Postweighting, all baseline covariates were well balanced between the TMAO and non‐TMAO groups (all SMD < 0.1; Supporting Table 4). The weighted TMAO subgroup approximated 1700 participants. Table 3 presents weighted baseline characteristics stratified by TMAO quartiles, with significant differences across quartiles in age, sex, smoking, alcohol consumption, hypertension, diabetes, and multiple metabolic and inflammatory parameters (all *p* < 0.05).

**TABLE 3:** Baseline characteristics of participants weighted by inverse probability weighting, stratified by TMAO quartiles.

	All (weighted *n* = 1701)	Q1 (weighted *n* = 444)	Q2 (weighted *n* = 443)	Q3 (weighted *n* = 418)	Q4 (weighted *n* = 397)	*p*
Age (years)	42.0 (24.0)	41.0 (21.0)	42.0 (24.0)	47.0 (30.0)	38.0 (15.0)	< 0.001
Male (%)	666 (39.2%)	175 (39.4%)	152 (34.3%)	157 (37.6%)	182 (46.0%)	0.006
BMI (kg/m^2^)	23.6 (4.3)	23.3 (4.2)	24.0 (4.6)	23.8 (4.2)	24.2 (4.4)	0.115
WHR	0.8 (0.1)	0.8 (0.1)	0.8 (0.1)	0.9 (0.1)	0.9 (0.2)	0.775
Smoker (%)	160 (9.4%)	28 (6.3%)	45 (10.2%)	39 (9.3%)	48 (12.1%)	0.033
Alcohol drinker (%)	310 (18.2%)	68 (15.3%)	60 (13.5%)	83 (19.9%)	99 (25.0%)	< 0.001
Hypertension	341 (20.1%)	98 (22.1%)	117 (26.4%)	90 (21.5%)	36 (9.1%)	< 0.001
Diabetes	33 (1.9%)	5 (1.1%)	6 (1.4%)	3 (0.7%)	19 (4.8%)	< 0.001
Red meat	340 (20.0%)	94 (21.2%)	62 (14.0%)	97 (23.2%)	87 (22.0%)	0.394
WBC (× 10^9^/L)	5.8 (2.1)	5.6 (2.3)	6.0 (2.4)	5.9 (1.8)	5.6 (1.5)	0.313
Lymphocyte	1.9 (0.7)	1.9 (0.7)	1.9 (0.7)	1.9 (0.6)	1.9 (0.2)	0.037
Neutrophil (× 10^9^/L)	3.5 (1.6)	3.4 (1.7)	3.5 (1.8)	3.5 (1.4)	3.2 (1.2)	0.266
BAS%	0.3 (0.3)	0.3 (0.3)	0.3 (0.3)	0.3 (0.2)	0.4 (0.2)	0.035
EOS%	1.6 (1.9)	1.5 (1.5)	1.4 (2.4)	1.7 (1.8)	2.8 (3.9)	0.028
NLR	1.8 (0.8)	1.8 (0.9)	1.8 (1.1)	1.9 (0.7)	1.6 (0.4)	0.005
LMR	7.2 (2.9)	7.1 (2.9)	7.5 (3.4)	7.1 (2.6)	6.6 (1.5)	0.023
ESR (mm/h)	4.0 (6.0)	1.8 (0.9)	1.8 (1.1)	1.9 (0.7)	1.6 (0.4)	0.036
TG (mmol/L)	1.1 (0.9)	1.0 (0.9)	1.1 (0.9)	1.1 (0.8)	1.2 (0.8)	0.028
TC (mmol/L)	4.6 (1.2)	4.4 (1.2)	4.7 (1.1)	4.7 (1.1)	4.6 (1.8)	0.008
HDL‐C (mmol/L)	1.4 (0.5)	1.4 (0.4)	1.4 (0.5)	1.4 (0.5)	1.4 (0.3)	0.120
LDL‐C (mmol/L)	2.8 (1.0)	2.8 (0.9)	2.7 (1.1)	2.9 (0.9)	2.6 (0.8)	0.013
GGT (U/L)	17.0 (13.0)	16.0 (14.0)	16.0 (12.0)	18.0 (11.0)	22.0 (44.7)	0.011
ALT (U/L)	15.0 (11.0)	14.7 (11.0)	15.0 (12.0)	14.0 (9.0)	15.0 (17.5)	< 0.001
TBIL (μmol/L)	10.8 (6.3)	11.9 (6.6)	11.0 (6.2)	10.3 (6.5)	10.3 (3.9)	0.187
Scr (μmol/L)	62.0 (16.0)	63.0 (17.0)	62.0 (15.0)	63.0 (18.0)	65.4 (19.7)	0.081
BUN (mmol/L)	4.1 (1.4)	3.9 (1.3)	4.1 (1.2)	4.4 (1.6)	4.5 (2.5)	< 0.001
UA (μmol/L)	276.0 (109.0)	263.0 (111.6)	274.0 (112.2)	282.0 (110.5)	291.0 (80.0)	< 0.001
*H. pylori* infection	982 (57.8%)	250 (56.30%)	244 (55.1%)	242 (57.9%)	246 (62.1%)	0.189
Plaque formation	817 (48.1%)	177 (39.90%)	199 (45.0%)	218 (52.2%)	223 (56.2%)	< 0.001

*Note:* TMAO, trimethylamine‐N‐oxide. Inverse probability weighting (IPW) was applied to adjust for selection bias. Quartiles of plasma TMAO were weighted to represent the full cohort.

### 3.4. Association Between TMAO and Carotid Plaque

Table 4 details the association between TMAO levels and carotid plaque in the weighted TMAO subgroup. In the unadjusted model, higher TMAO quartiles were associated with an increased risk of carotid plaque (Q4 vs. Q1: OR 1.88, 95% CI 1.43–2.48, *p* < 0.001). After adjusting for demographic characteristics and conventional cardiovascular risk factors (Multivariable Model 2c), this association remained significant in both TMAO (Q3) and TMAO (Q4). Notably, model stability was compromised with the inclusion of additional candidate variables (Multivariable Model 4), requiring further variable adjustment. The TMAO (Q3) association remained significant, but it attenuated substantially and lost statistical significance after additional age adjustment (Multivariable Model 5f). In contrast, *H. pylori* infection maintained a consistently significant association with carotid plaque across all models (OR: 1.73–2.59, all *p* < 0.001), highlighting the robustness of the primary findings.

**TABLE 4:** Weighted multivariable analysis of the association between plasma TMAO level, *H. pylori* infection, and carotid plaque formation.

Model	OR (95% CI)	*p* ^∗^	AUC (95% CI)	H‐L *p* ^#^	EPV
Crude model			0.593 (0.549, 0.636)	0.047	—
TMAO (Q1)	1.00 (referent)				
TMAO (Q2)	1.25 (0.95, 1.63)	0.111			
TMAO (Q3)	1.63 (1.24, 2.14)	< 0.001			
TMAO (Q4)	1.88 (1.43, 2.48)	< 0.001			
*H. pylori*	1.73 (1.42, 2.11)	< 0.001			
Multivariable Model 1			0.629 (0.587, 0.672)	0.004	163.4
TMAO (Q1)	1.00 (referent)				
TMAO (Q2)	1.23 (0.94, 1.61)	0.140			
TMAO (Q3)	1.64 (1.25, 2.16)	< 0.001			
TMAO (Q4)	1.81 (1.37, 2.40)	< 0.001			
*H. pylori*	1.78 (1.46, 2.17)	< 0.001			
Multivariable Model 2			0.683 (0.643, 0.724)	0.045	74.3
TMAO (Q1)	1.00 (referent)				
TMAO (Q2)	1.24 (0.933, 1.65)	0.139			
TMAO (Q3)	1.61 (1.21, 2.15)	0.001			
TMAO (Q4)	1.72 (1.28, 2.31)	< 0.001			
*H. pylori*	1.75 (1.42, 2.15)	< 0.001			
Multivariable Model 3			0.755 (0.705, 0.806)	0.048	51.1
TMAO (Q1)	1.00 (referent)				
TMAO (Q2)	1.53 (1.05, 2.22)	0.027			
TMAO (Q3)	2.01 (1.37, 2.95)	< 0.001			
TMAO (Q4)	0.48 (0.19, 1.25)	0.134			
*H. pylori*	2.39 (1.76, 3.25)	< 0.001			
Multivariable Model 4			0.755 (0.705, 0.806)	0.240	58.4
TMAO (Q1)	1.00 (referent)				
TMAO (Q2)	1.51 (1.04, 2.18)	0.031			
TMAO (Q3)	1.97 (1.35, 2.88)	< 0.001			
TMAO (Q4)	0.48 (0.19, 1.23)	0.125			
*H. pylori*	2.39 (1.76, 3.25)	< 0.001			
Multivariable Model 5			0.948 (0.926, 0.970)	0.012	54.5
TMAO (Q1)	1.00 (referent)				
TMAO (Q2)	1.72 (0.97, 3.05)	0.063			
TMAO (Q3)	1.76 (0.98, 3.16)	0.058			
TMAO (Q4)	0.57 (0.15, 2.14)	0.404			
*H. pylori*	2.59 (1.59, 4.20)	< 0.001			

*Note:* Crude model: Odds ratios and 95% CI were estimated by *H. pylori* infection and plasma TMAO levels. Multivariable Model 1 was adjusted for demographic factors (sex, smoking status, alcohol consumption). Multivariable Model 2 was additionally adjusted for traditional cardiovascular risk factors (BMI, hypertension, FBG, LDL‐C, HDL‐C, and TG). Multivariable Model 3 was additionally adjusted for additional candidate variables that had a *p* < 0.10 in the univariate analysis (NLR, ESR, GGT, TBIL, SCR). Due to the poor calibration of Model 3, Multivariable Model 4 was additionally adjusted for variables (ESR, GGT, TBIL) based on Model 2. Multivariable Model 5 was additionally adjusted for age. All models were weighted using inverse probability weighting.*p* ^∗^ < 0.025 were considered statistically significant to account for multiple comparisons across TMAO quartiles.

## 4. Discussion

In this cross‐sectional study, we examined the associations among *Helicobacter pylori* infection, plasma TMAO levels, and carotid plaque in a health screening population. We found that *H. pylori* infection was associated with carotid plaque after adjustment for multiple covariates, whereas the association between TMAO and carotid plaque was markedly attenuated after adjustment for age. In addition, no significant association was observed between *H. pylori* infection and circulating TMAO levels. Importantly, due to the cross‐sectional design, the temporal sequence among these variables cannot be determined, and causal inferences should be avoided.

The association between *H. pylori* infection and carotid plaque observed in this study is broadly consistent with previous epidemiological evidence suggesting a link between chronic infection and atherosclerotic disease [10, 11]. A growing body of literature has proposed that *H. pylori* infection may contribute to atherosclerosis through systemic inflammation, immune activation, and endothelial dysfunction [9]. Chronic infection has been associated with persistent low‐grade inflammation, which is increasingly recognized as an important contributor to the development and progression of atherosclerosis [16]. In line with these observations, we found that individuals with *H. pylori* infection exhibited higher levels of white blood cells and related inflammatory indices, which may reflect an enhanced inflammatory state.

White blood cells and their subtypes are well‐established markers of inflammation and have been associated with cardiovascular risk and vascular damage [17, 18]. Previous studies have demonstrated that elevated white blood cell counts are linked to arterial stiffness and atherosclerotic changes [19, 20]. Therefore, the observed elevation of inflammatory markers in *H. pylori*‐positive individuals may provide indirect support for a potential association between infection and vascular pathology. However, it should be emphasized that, given the observational nature of the present study, it remains unclear whether *H. pylori* infection directly contributes to plaque formation or whether the observed relationship is driven by shared risk factors or residual confounding. Indeed, previous large‐scale analyses have reported inconsistent findings regarding the association between *H. pylori* infection and cardiovascular outcomes [21], highlighting the need for cautious interpretation.

With respect to TMAO, although higher levels were associated with carotid plaque in unadjusted analyses, this association was substantially attenuated after adjustment for age, suggesting that age is a key confounding factor. TMAO is a gut microbiota‐derived metabolite generated from dietary precursors such as choline and carnitine, and has been implicated in CVD in both experimental and epidemiological studies [12, 22]. Several studies have reported positive associations between circulating TMAO levels and adverse cardiovascular outcomes, including atherosclerosis and mortality [23, 24]. However, TMAO levels are influenced by multiple factors, including diet, renal function, and gut microbiota composition, all of which may vary substantially with age.

Aging is associated with changes in renal function, which may reduce TMAO clearance, as well as alterations in the gut microbiota composition and metabolic regulation [25, 26]. These factors may lead to elevated circulating TMAO levels independent of direct pathological effects. In this context, the attenuation of the TMAO–plaque association after age adjustment observed in our study suggests that TMAO may primarily reflect age‐related physiological processes rather than serving as an independent determinant of carotid atherosclerosis. This interpretation is consistent with emerging evidence linking TMAO to age‐related conditions such as cognitive decline, inflammation, and frailty [27]. Nevertheless, the precise role of TMAO in CVD remains an area of ongoing debate, and further studies are required to disentangle its potential causal and noncausal contributions.

Several limitations should be acknowledged. First, the cross‐sectional design precludes causal inference and limits assessment of temporal relationships. Second, residual confounding remains a concern: important determinants of TMAO and atherosclerosis (detailed dietary patterns, antibiotic exposure, and medication use such as statins) were not systematically recorded, and renal function was only partially adjusted for. Third, *H. pylori* infection was assessed using a serological assay, which cannot distinguish between current and past infection and may introduce misclassification. Fourth, carotid plaque assessment was based on standardized ultrasonography, but interobserver variability was not formally evaluated. Finally, although IPW and model evaluation metrics were applied, the potential for overfitting remains, and interaction analyses, validation, and assessment of the incremental predictive value of TMAO were not performed.

## 5. Conclusion

In summary, our findings suggest that *H. pylori* infection is associated with carotid plaque in this health screening population, whereas the association between TMAO and carotid plaque appears to be largely explained by age. These results highlight potential links among infection, metabolism, and atherosclerosis but should be interpreted as hypothesis‐generating. Further prospective studies with detailed assessment of diet, medication use, and microbiome composition are required to clarify the underlying relationships and potential mechanisms.

## Author Contributions

Fang Liu, Lina Zheng, Xia Cao, and Pei Peng designed the research. Yaoze Li and Xiangdong Xu performed formal analysis. Yihan Zhou, Yun Zhang, Yue Wang and Xue Sun collected clinical data. Jiangman Zhao, and Pei Peng wrote the manuscript.

## Funding

This study was supported by the Joint Project of Henan Medical Science and Technology Research Plan in 2023 (LHGJ20230797).

## Disclosure

All authors read and approved the final manuscript.

## Ethics Statement

Ethical approval was obtained from the Kaifeng Central Hospital Ethics Committee (2022ky‐024), and written informed consent was collected from all participants.

## Conflicts of Interest

The authors declare no conflicts of interest.

## Supporting Information

Additional supporting information can be found online in the Supporting Information section.

## Supporting information


**Supporting Information** Supporting Table 1. Baseline characteristics of participants with and without TMAO measurement. Supporting Table 2. Univariate logistic regression analysis of factors associated with carotid plaque formation. Supporting Table 3. Collinearity diagnostics for multivariable models. Supporting Table 4. Covariate balance before and after inverse probability weighting.

## Data Availability

The data that support the findings of this study are available on request from the corresponding author. The data are not publicly available due to privacy or ethical restrictions.
