# Supplementary material for: Relationship Between Helicobacter pylori, Trimethylamine‐N‐Oxide, and Carotid Plaque Formation Among Adult Health Checkup Population in China
Source: Cardiol Res Pract. 2026 Oct 8;2026:5468109. doi: 10.1155/crp/5468109 (PMC13647107; doi:10.1155/crp/5468109)
Supplement: Supplementary file 1 [file CRP-2026-5468109-s001.docx]

**Supplementary Table 1. Baseline characteristics of participants with and without TMAO measurement**

| **Clinical features** | **Participants with TMAO detection**  **(n=726)** | **Participants without TMAO detection**  **(n=1053)** | **P value** |
| --- | --- | --- | --- |
| Age, years | 48.00 (29.00) | 43.00 (21.75) | **<0.001** |
| Male, % | 287 (39.53%) | 414 (39.32%) | 0.927 |
| BMI (kg/m2) | 23.24 (3.99) | 23.71 (4.64) | **<0.001** |
| WHR | 0.84 (0.11) | 0.84 (0.12) | 0.326 |
| Smoker, % | 74 (10.19%) | 90 (8.55%) | 0.238 |
| Alcohol drinker, % | 143 (19.70%) | 184 (17.47%) | 0.234 |
| Hypertension | 101 (13.91%) | 239 (22.70%) | **<0.001** |
| Diabetes | 21 (2.89%) | 16 (1.52%) | **0.046** |
| WBC, ×10^9^/L | 5.74 (2.05) | 5.70 (1.92) | 0.470 |
| Lymphocyte, ×10^9^/L | 1.93 (0.78) | 1.90 (0.70) | 0.226 |
| Neutrophil, ×10^9^/L | 3.42 (1.54) | 3.30 (1.43) | 0.568 |
| BAS% | 0.30 (0.30) | 0.30 (0.30) | 0.867 |
| EOS% | 1.60 (1.70) | 1.60 (1.70) | 0.389 |
| NLR | 1.79 (0.90) | 1.78 (0.89) | 0.759 |
| LMR | 7.27 (3.17) | 7.26 (3.33) | 0.536 |
| ESR, mm/h | 5.00 (7.00) | 4.00 (6.00) | 0.586 |
| TG, mmol/L | 1.21 (0.96) | 1.10 (0.90) | **<0.001** |
| TC, mmol/L | 4.63 (1.19) | 4.60 (1.19) | 0.713 |
| HDL-C, mmol/L | 1.39 (0.40) | 1.39 (0.40) | 0.157 |
| LDL-C, mmol/L | 2.73 (1.02) | 2.76 (0.99) | **<0.001** |
| GGT, U/L | 18.00 (14.00) | 18.00 (16.00) | 0.219 |
| ALT, U/L | 15.00 (11.00) | 16.00 (12.25) | 0.154 |
| TBIL, umol/ L | 10.75 (6.20) | 10.60 (6.00) | 0.113 |
| Scr, µmol/ L | 63.00 (16.00) | 63.00 (18.00) | 0.845 |
| BUN, mmol/ L | 4.30(1.40) | 4.30 (1.42) | 0.219 |
| UA, umol/L | 283.00 (110.00) | 281.00 (107.00) | 0.712 |
| H. pylori infection | 442 (60.9%) | 631 (59.9%) | 0.685 |
| Plaque formation | 415 (57.2%) | 449 (42.6%) | **<0.001** |

**Supplementary table 2. Univariate logistic regression analysis of factors associated with carotid plaque formation**

| **Index** | **B** | **S.E.** | **Wald** | **df** | **P** | **OR (95% CI)** |
| --- | --- | --- | --- | --- | --- | --- |
| Age, years | 0.165 | 0.007 | 502.838 | 1 | **＜0.001** | 1.179(1.162,1.196) |
| Male,% | -0.478 | 0.098 | 23.951 | 1 | **＜0.001** | 0.620（0.512,0.751） |
| BMI (kg/m2) | 0.000 | 0.000 | 0.296 | 1 | **0.587** | 1.000（0.999,1.000） |
| Smoker, % | -0.755 | 0.171 | 19.405 | 1 | **＜0.001** | 0.470(0.336,0.658) |
| Alcohol drinker, % | -0.379 | 0.123 | 9.453 | 1 | **0.002** | 0.685(0.538,0.872) |
| Hypertension | 0.148 | 0.121 | 1.491 | 1 | 0.222 | 1.159(0.914,1.469) |
| Diabetes | -0.684 | 0.348 | 3.874 | 1 | **0.049** | 0.504(0.255,0.997) |
| WBC, ×10^9^/L | 0.000 | 0.003 | 0.002 | 1 | 0.967 | 1.000(0.994,1.006) |
| Lymphocyte, ×10^9^/L | 0.050 | 0.081 | 0.374 | 1 | 0.541 | 1.051(0.896,1.233) |
| BAS% | 0.141 | 0.271 | 0.272 | 1 | 0.602 | 1.152(0.677,1.959) |
| EOS% | 0.033 | 0.028 | 1.388 | 1 | 0.239 | 1.034(0.978,1.093) |
| NLR | 0.081 | 0.038 | 4.516 | 1 | **0.034** | 1.084(1.006,1.168) |
| LMR | 0.021 | 0.018 | 1.418 | 1 | 0.234 | 1.022(0.986,1.058) |
| ESR, mm/h | 0.060 | 0.011 | 32.227 | 1 | **＜0.001** | 1.062(1.040,1.085) |
| TG, mmol/L | 0.219 | 0.049 | 20.090 | 1 | **＜0.001** | 1.245(1.131,1.369) |
| TC, mmol/L | 0.475 | 0.057 | 69.453 | 1 | **＜0.001** | 1.608(1.438,1.799) |
| HDL-C, mmol/L | -0.473 | 0.155 | 9.307 | 1 | **0.002** | 0.623(0.460,0.845) |
| LDL-C, mmol/L | 0.001 | 0.007 | 0.021 | 1 | 0.886 | 1.001(0.987,1.015) |
| GGT, U/L | 0.006 | 0.002 | 8.550 | 1 | **0.003** | 1.006(1.002,1.010) |
| ALT, U/L | 0.003 | 0.002 | 1.289 | 1 | 0.256 | 1.003(0.998,1.008) |
| TBIL, umol/L | 0.025 | 0.009 | 7.967 | 1 | **0.005** | 1.025(1.008,1.043) |
| Scr, µmol/L | 0.014 | 0.004 | 14.890 | 1 | **＜0.001** | 1.014(1.007,1.021) |
| BUN, mmol/L | -0.001 | 0.005 | 0.059 | 1 | 0.809 | 0.999(0.990,1.008) |
| UA, umol/L | 0.001 | 0.001 | 1.107 | 1 | 0.293 | 1.001(0.999,1.002) |
| *H. pylori* infection | -0.549 | 0.098 | 31.270 | 1 | **＜0.001** | 1.731（1.428,2.099） |

**Supplementary table 3. Collinearity diagnostics for multivariable models**

| **Index** | **Tolerance** | **VIF^a^** | **VIF^b^** |
| --- | --- | --- | --- |
| *H. pylori* infection | 6.028 | 1.012 | 1.011 |
| Male,% | 1.404 | 2.682 | 2.676 |
| Age, years | 34.103 | 1.199 | 1.188 |
| Smoker, % | 2.89 | 1.961 | 1.961 |
| Alcohol drinker, % | -1.842 | 2.401 | 2.401 |
| BMI (kg/m^2^) | -3.032 | 1.477 | 1.462 |
| TG, mmol/L | 1.071 | 3.386 | 1.54 |
| TC, mmol/L | -2.004 | 15.867 | - |
| HDL-C, mmol/L | -0.712 | 3.919 | 1.468 |
| LDL-C, mmol/L | 3.377 | 12.709 | 1.175 |
| Hypertension | -0.316 | 1.008 | 1.008 |
| Diabetes | 0.873 | 1.015 | 1.015 |
| NLR | -0.489 | 1.032 | 1.027 |
| ESR, mm/h | -0.681 | 1.237 | 1.236 |
| GGT, U/L | 2.252 | 1.499 | 1.499 |
| TBIL, umol/L | -0.502 | 1.146 | 1.144 |
| Scr, µmol/L | -0.65 | 1.617 | 1.616 |

Notes: VIF, variance inflation factor. VIF > 5 indicates significant multicollinearity.

VIF^a^ calculated with all candidate variables. VIF^b^ calculated after removing total cholesterol (TC) due to high collinearity with LDL-C and HDL-C.

**Supplementary table 4. Covariate balance before and after inverse probability weighting**

| **Index** | **before** | | | **after** | | |
| --- | --- | --- | --- | --- | --- | --- |
|  | **with TMAO detection (n=728)** | **without TMAO detection(n=1051)** | **SMD** | **with TMAO detection(n=1701)** | **without TMAO detection(n=1684)** | **SMD** |
| Male,% | 37.0 | 42.0 | 0.10 | 39.0 | 40.0 | 0.020 |
| Age, years | 45.11 ± 14.31 | 49.89 ± 14.58 | ****0.33**** | 46.98 ± 14.49 | 47.09 ± 14.79 | 0.008 |
| BMI (kg/m2) | 23.88 ± 3.53 | 23.73 ± 3.18 | 0.04 | 23.81 ± 3.27 | 23.81 ± 3.45 | 0.000 |
| Smoker, % | 8.0 | 12.0 | **0.13** | 9.0 | 9.0 | 0.000 |
| Alcohol drinker, % | 16.0 | 21.0 | **0.13** | 18.0 | 18.0 | 0.000 |
| Hypertension | 22.0 | 14.0 | **0.21** | 20.0 | 19.0 | 0.025 |
| Diabetes | 1.0 | 3.0 | **0.14** | 2.0 | 2.0 | 0.000 |
| TG, mmol/L | 1.447 ± 1.459 | 1.506 ± 1.106 | 0.04 | 1.466 ± 1.212 | 1.460 ± 1.376 | 0.005 |
| HDL-C, mmol/L | 1.424 ± 0.309 | 1.358 ± 0.301 | **0.22** | 1.407 ± 0.323 | 1.403 ± 0.301 | 0.013 |
| LDL-C, mmol/L | 2.806 ± 0.728 | 2.876 ± 0.747 | 0.09 | 2.831 ± 0.728 | 2.829 ± 0.730 | 0.003 |
| BUN, mmol/L | 4.437 ± 1.202 | 4.643 ± 2.555 | 0.10 | 4.511 ± 1.961 | 4.495 ± 1.230 | 0.010 |
| Plaque formation | 42.0 | 58.0 | ****0.32**** | 48.0 | 49.0 | 0.020 |

Notes: SMD, standardized mean difference. SMD < 0.1 indicates adequate balance after weighting.
